# Applying Research on Cultural Narratives About Living With Dementia to Social Policy and Practice

**DOI:** 10.1093/geroni/igaf122.969

**Published:** 2025-12-31

**Authors:** Nancy Berlinger, Nancy Berlinger

## Abstract

This symposium presents a 2025 report on cultural narratives about dementia, funded by the National Endowment for the Humanities. Cultural narratives are stories expressed through metaphors and other representations that circulate through social groups to make meaning out of observations. By shaping common knowledge, cultural narratives influence social policy and practice concerning responses to societal needs. Papers will show how recognition of cultural narratives, practices of narrative repair, and the generation of new narratives are integral to supporting people with dementia in a society and communities. The first paper analyzes selected cultural narratives, showing how they convey ideas about improving dementia care. It presents recommendations for improving policy development. The second paper draws on findings from community-based participatory research (CBPR) with North American Indigenous communities that reveal community-level narratives about aging and dementia. It explains strategies that respect these narratives. The third paper considers the experiences of older Black women in the context of elevated risk of dementia, precursor conditions, later-life economic precarity, and caregiving. It suggests narrative repair strategies to better represent and support these experiences. The fourth paper explores the concept of brain health as it applies to Black men, a group at elevated risk of dementia and precursor conditions. It draws on findings from CBPR and community health initiatives to consider new narratives about brain health that include Black men. The fifth paper offers insights from research and practice on an innovative dementia-friendly initiative for congregations. It explains how dementia-friendliness narratives can evolve from awareness to inclusion.

